# The Effect of Corporate Social Responsibility on Workplace Safety: The Significance of Employees’ Moral Identity

**DOI:** 10.3390/bs14060429

**Published:** 2024-05-22

**Authors:** Yunsook Hong, Taewoo Roh

**Affiliations:** 1 College of Business Administration, University of Ulsan, Ulsan 44610, Republic of Korea; yhong@ulsan.ac.kr; 2 School of International Studies, Hanyang University, Seoul 04763, Republic of Korea

**Keywords:** corporate social responsibility, moral identity, job stress, organizational identification, workplace safety, moderated mediation model

## Abstract

Corporate social responsibility (CSR) has gained significant attention from researchers and practitioners due to its potential impact on employee attitudes and behaviors. This study investigates the influence of CSR on employee safety behavior, examining the mediating effects of job stress and organizational identification, as well as the moderating role of moral identity. We conducted a three-wave time-lagged survey among 236 South Korean employees. Participants were recruited through a research company using stratified random sampling. Data were collected at three time points, each separated by a 4–5-week interval. Structural equation modeling (SEM) was used to test the hypothesized relationships. The findings support all of the hypothesized relationships. CSR was positively related to safety behavior and negatively related to job stress. Job stress was negatively related to organizational identification, which in turn was positively related to safety behavior. The sequential mediation of job stress and organizational identification was significant. Moral identity moderated the relationship between CSR and job stress, such that the negative effect of CSR on job stress was stronger for employees with a high moral identity. This study contributes to the CSR literature by examining the impact of CSR on safety behavior and the underlying mechanisms and boundary conditions of this relationship. The findings suggest that organizations can promote employee safety behavior by implementing CSR initiatives, which can reduce job stress and enhance organizational identification. Organizations should also consider the role of employees’ moral identity when designing and communicating CSR initiatives.

## 1. Introduction

Recently, the importance of corporate ethics has become a critical concern for businesses, prompting comprehensive examination by scholars and practitioners in the field of corporate social responsibility (CSR) [1,2,3]. CSR embodies a plethora of interpretations and can be comprehensively defined as an amalgamation of strategies and practices deployed by corporations to ameliorate their economic, societal, and environmental performance while concurrently catering to the diverse requisites of stakeholders, including employees, customers, local communities, governments, and the environment [4,5]. Despite an abundance of research investigating the repercussions of CSR on organizational performance, the outcomes have been equivocal [6,7,8,9]. Some studies propose that CSR initiatives function as a ‘strategic investment,’ endowing companies with a competitive edge [10,11,12]. Conversely, certain scholars argue that allocating resources to social responsibilities might translate into a “cost” that could impede operational efficacy [13,14]. To bridge the gap between these conflicting perspectives, scholars have explored various fundamental mechanisms and contextual factors that influence how CSR initiatives impact a company’s various performances [4,6,7,13,15,16,17,18].

In spite of abundant studies into the effects of CSR on organizational performance, several lacunae remain in the prevailing literature [6,8,9]. First, the majority of existing CSR research has focused on the implications of corporate social responsibility on macro-level performance, including financial performance, customer loyalty, and the prestige of the firm. However, these studies have primarily neglected individual-level performance, including the perceptions, attitudes, and behaviors of firm members [6,8]. Considering that employees are the primary agents responsible for planning and executing CSR activities within an organization and translating ethical conduct (e.g., CSR) into organizational outcomes, it is crucial to examine employee responses to CSR initiatives. As a result, future research should strive to bridge these gaps and shed light on the complex interplay between CSR and its diverse organizational and individual-level effects.

Second, although some earlier research has explored the consequences of corporate social responsibility on members, most investigations have concentrated on individuals’ perceptions and attitudes. These include identification with their organization, organizational trust, the meaningfulness of their work, their intrinsic motivation, and employee engagement [19,20,21,22,23,24,25,26]. On the other hand, employee actions have received comparatively less attention [6,8,9,27]. Although perceptions and attitudes are crucial individual-level outcomes within a company, they tend to ultimately manifest as behaviors [6,8,27]. Hence, we postulate that employee conduct exhibits a more robust affiliation with diverse organizational results compared to cognitions and attitudes, rendering it imperative to scrutinize the effect of CSR on employee behavior.

Third, prior research has inadequately probed into the impact of CSR on workplace safety [6,8,27]. As delineated by Burke and his associates [28], safety performance encompasses actions executed in most professions that safeguard the health and safety of employees, customers, and the public. Employees’ safety behavior is deemed a pivotal contributing element to workplace mishaps and injuries [29], and its significance has been emphasized by researchers, particularly in the aftermath of the COVID-19 pandemic, as it plays an integral role in ensuring both customer and organizational health and safety during major health crises [27,30]. Considering the importance of safety behavior within organizations, it is vital to investigate the influence of CSR on it.

Fourth, pertinent to the third research gap in the existing CSR literature, there has been insufficient exploration of the mediators and moderators that affect the associations between CSR and safety conduct [6,8,27]. As identifying the mediators and moderators can offer a holistic understanding of this association [6,8], it is crucial to explore the factors that mediate and moderate the links between CSR and safety conduct.

To address these research gaps, the current study delves into the underlying procedures (mediators) and their situational variables (moderators) in corporate social responsibility–safety behavior associations. Specifically, drawing on previous research on CSR, moral identity, job stress, organizational identification, and safety behavior, we propose that employees’ job stress and organizational identification may sequentially mediate the relationship between CSR and safety behavior, and that their moral identity would positively moderate the CSR–job stress link.

To delve into these hypotheses, this study applies structural equation modeling (SEM) to establish a moderated mediation model relying on tripartite time-lagged data acquired from 236 Korean employees. This paper may contribute significantly to the literature on CSR and workplace safety in the following ways. First and foremost, our investigation seeks to elucidate the ramifications of CSR on employee safety behavior, which has thus far remained relatively uncharted in the extant literature. Recognizing the immense pertinence of this relationship for both scholarly pursuits and industry practitioners, we aim to expand the breadth of understanding in this domain, thus offering invaluable insights for organizations to optimize employee safety and well-being.

Second, this study delves into the underlying mechanisms (mediators) and situational factors (moderators) that preside over the nexus between CSR and safety behavior. In particular, we pinpoint job stress and employee organizational identification as sequential mediators, thereby illuminating the intricate interplay of variables that sculpt the CSR–safety behavior connection. This refined understanding permits a more all-encompassing appreciation of the dynamics in action and proffers guidance for interventions to ameliorate workplace safety outcomes.

Third, we emphasize the pivotal role of employees’ moral identity in the aforesaid relationships. Our empirical evidence substantiates the notion that moral identity operates as a positive moderator, amplifying the decreasing effect of CSR on job stress. This revelation accentuates the necessity of cultivating an ethical environment within organizations, as it can bolster the potency of CSR initiatives in fostering employee well-being and safety.

Fourth, on a methodological level, our research endeavors to redress the shortcomings endemic to cross-sectional designs by adopting a three-wave, time-lagged data collection strategy. This rigorous methodology empowers us to ascertain the temporality and causality of relationships between the variables under scrutiny, thereby enhancing the validity and generalizability of our conclusions.

The objectives of this study are threefold:

1. To examine the direct relationship between CSR and employee safety behavior;
2. To investigate the sequential mediating effects of job stress and organizational identification on the relationship between CSR and employee safety behavior;
3. To explore the moderating role of moral identity on the relationship between CSR and job stress.

In summation, this paper constitutes a momentous addition to the CSR and safety behavior literature by unveiling the hitherto unexplored associations between these constructs, disentangling the underlying mediators and moderators, and utilizing a robust methodological approach. These insights serve to enrich our comprehension of the dynamics at work and provide pragmatic guidance for organizations endeavoring to bolster employee safety and well-being via the implementation of corporate social responsibility.

## 2. Theory and Hypotheses

### 2.1. Corporate Social Responsibility and Safety Behavior

Despite limited research exploring the influence of corporate social responsibility on the safety behavior of members [6,8,27], it can be inferred from the social exchange theory that CSR initiatives can encourage such behavior [31,32]. The theory of social exchange proposes that when one entity offers assistance or benefits to another, the beneficiary is predisposed to respond in kind [33,34]. As primary stakeholders within an organization, employees are directly and indirectly influenced by the organization’s CSR undertakings [6,8,27]. Consequently, they are likely to experience a sense of indebtedness to their organization, ultimately manifesting their appreciation through constructive actions. These may encompass the display of safe occupational conduct [6,8,27,35]. This theory suggests that when employees perceive their organization as socially responsible and committed to their well-being, they are more likely to reciprocate by engaging in positive behaviors, such as adhering to safety guidelines and participating in safety-related activities [36,37].

Previous studies have investigated the relationship between CSR and various aspects of employee behavior, including safety behavior. Hofmann et al. [38] found that employees’ perceptions of their organization’s commitment to safety, which can be viewed as a form of CSR, were positively related to safety citizenship behavior. Similarly, De Roeck and Maon [39] argued that CSR initiatives can foster a sense of organizational pride and identification among employees, leading to increased safety compliance and participation.

**Hypothesis 1:**  *CSR activities will increase the degree of safe behavior of employees.*

### 2.2. CSR and Job Stress

In the current study, it is suggested that CSR may decrease employees’ job stress. Notably, few studies have investigated the association between CSR and job stress [6,8]. Considering that job stress has significantly impacted employees’ well-being, performance, and turnover intentions, a deeper understanding of CSR–job stress links is vital for the contemporary workforce. This study attempts to explain these links by employing relevant theoretical backgrounds. Together, the job demands–resources (JD-R) model [40,41], social identity theory (SIT) [42,43], and conservation of resources (COR) theory [44,45] should provide a comprehensive understanding of how CSR initiatives can potentially alleviate job stress among employees.

The JD-R model serves as a valuable tool to elucidate CSR–job stress links. This model posits that employee well-being and job stress stem from the equilibrium between job demands (e.g., workload, time pressure) and job resources (e.g., social support, autonomy) [40,41]. Within this framework, CSR initiatives can be regarded as organizational resources that potentially diminish employees’ job stress by equipping them with additional tools to manage the demands of their jobs [40,41]. According to this model, job resources, such as organizational support and a positive work environment, can buffer the negative impact of job demands on employee well-being. CSR initiatives can be viewed as a form of organizational support that helps employees cope with job demands and reduces their experience of job stress [46].

According to the SIT, employees are more inclined to identify with their organizations if they actively engage in CSR activities. From a member’s perspective, such ethical activities may appear to align with the employee’s values and contribute to boosting a favorable self-image at the workplace [42,43]. As a result, the enhanced social identity of the employee may facilitate a sense of pride as well as a perception of unity with the socially responsible organization [42,43]. These impressions and feelings ultimately lower the employee’s perceived job stress levels.

The COR theory postulates that employees tend to endeavor to acquire, maintain, and protect valued resources, encompassing both personal and organizational resources [44,45]. From this perspective, CSR activities are regarded as valuable organizational resources that employees can draw upon to cope with their job stress. Individuals strive to protect and acquire such organizational resources, which eventually lowers their job stress [47].

Research has demonstrated that CSR initiatives can have a positive impact on employee well-being, including reduced job stress. Flammer and Luo [48] found that firms with higher levels of CSR engagement had employees with lower levels of job stress and higher job satisfaction. Kim and Kim [47] also reported that employees’ perceptions of their organization’s CSR were negatively associated with work-related stress.

**Hypothesis 2:**  *CSR activities will decrease the degree of employee job stress.*

### 2.3. Job Stress and Organizational Identification

We suggest that a decrease in employee job stress would increase their organizational identification. Numerous studies have demonstrated a strong correlation between job-related stress experienced by employees and their attitudes within an organization. This includes their organizational identification [49,50,51,52,53]. Organizational identification refers to the perception of oneness with or belongingness to an organization, where an individual’s beliefs about their organization become self-referential [54,55]. Highly identified employees are likely to adopt the organization’s objectives as their own, striving for organizational profit maximization beyond personal interests. This can ultimately lead to positive outcomes such as increased job satisfaction and improved discretionary behavior and task performance [50,52,55,56].

The social exchange framework offers theoretical underpinnings for the job stress–organizational identification link. This perspective is centered around the concept of reciprocity, which suggests that if an individual or group provides something of value to another, the recipient feels obligated to repay them similarly [33,34,35]. Grounded in social exchange theory, it is likely that the quality of interaction between a member and the organization dictates the extent of identification with the organization [33,34]. Consistent with this theory, an individual employee experiencing significant job stress endeavors to maintain equilibrium between themselves and the organization, and occasionally attempts to reciprocate the uncomfortableness that originates from the job stress [34,49,50,51,53,54]. Due to the inherent power imbalance between employees and their organizations, employees may be unable to directly counter the discomfort stemming from job stress. Instead, they may respond against their organization in passive and indirect ways including worsening attitudes, such as diminished organizational identification. This reduction in organizational identification enables employees to regain a sense of psychological equilibrium [34,35,49,50,51,53,54,55].

In addition, the link between reduced job stress and increased organizational identification can be explained through social identity theory [57]. According to this theory, individuals derive a sense of self-worth and identity from their membership in social groups, including their work organizations. When employees experience lower levels of job stress, they are more likely to view their organization positively and identify with it more strongly [58]. Furthermore, reduced job stress can create a more positive work environment, which can foster a sense of belonging and identification among employees [59].

Studies have explored the relationship between job stress and organizational identification, with findings suggesting that reduced job stress can lead to increased organizational identification. Avanzi et al. [59] found that job stressors, such as role ambiguity and role conflict, were negatively related to organizational identification. Similarly, Van Knippenberg et al. [60] argued that job stress can hinder employees’ ability to identify with their organization.

**Hypothesis 3:**  *Decreased job stress will enhance employee organizational identification.*

### 2.4. Organizational Identification and Safety Behavior

We suggest that a greater amount of employee organizational identification may lead to a greater degree of safety-oriented behavior. Employees with a high level of organizational identification tend to feel more closely connected to their organization, perceive a greater sense of belonging, and ultimately accept its values and goals [50,52,54,55]. This sense of connection and belonging results in more positive attitudes toward the organization, and greater efforts toward achieving organizational objectives and success [56]. Numerous studies have demonstrated a close association between organizational identification and organizational citizenship behavior, as well as cooperative behavior [50,52,54,55].

Specifically, employee organizational identification would increase the extent of their behaviors aligning with the firm’s goals. It would also diminish the behaviors that contradict the organization’s values and success. Members who perceive themselves as identified with their organization are less likely to engage in deviant behavior that could harm it. Additionally, members may perceive that the success of their firm at least partially determines their success. Such members attempt to diminish behaviors that do not align with the firm’s objectives and support its success, since they understand that harmful actions such as unsafe behavior can harm both the organization and themselves [50,52,54,55]. Therefore, we propose that increased organizational identification can improve safety-oriented behavior, which will support the company’s success.

The relationship between organizational identification and safety behavior can be understood through the lenses of social identity theory [57] and social exchange theory [31]. When employees strongly identify with their organization, they are more likely to internalize the organization’s values and goals, including those related to safety [61]. Additionally, employees with high organizational identification may view engaging in safety behavior as a way to reciprocate the organization’s commitment to their well-being and to contribute to the organization’s success [62].

Research has investigated the impact of organizational identification on various aspects of employee behavior, including safety behavior. Wachter and Yorio [61] found that employees with higher levels of organizational identification were more likely to engage in safety compliance and participation behaviors. Lee et al. [62] also reported that organizational identification was positively associated with safety performance among construction workers.

**Hypothesis 4:**  *Increased organizational identification will enhance the degree of safety behavior.*

### 2.5. Sequential Mediation of Job Stress and Organizational Identification in the Corporate Social Responsibility–Safety Behavior Link

The current research conjectures that the association between CSR and safety behavior is sequentially mediated by job stress and organizational identification, predicated on the firmly established context–attitude–behavior framework [63,64]. This theoretical model asserts that the contextual and environmental variables of an organization, which include systems, practices, rules, and climates, wield significant sway over the attitudes and behaviors of its members.

As a critical contextual factor, CSR has the potential to shape employees’ attitudes, particularly with respect to job stress and organizational identification. These attitudinal shifts, in turn, can significantly impact employees’ actions, such as their engagement in workplace safety behavior. By delineating the sequential mediating effect of job stress and organizational identification, this study seeks to provide a more elaborate understanding of the underlying processes through which CSR influences employee safety behavior. In doing so, we aim to strengthen the theoretical underpinnings of the CSR–safety behavior link and offer valuable insights for organizations seeking to enhance workplace safety through CSR initiatives.

**Hypothesis 5:**  *Employees’ job stress and organizational identification will sequentially mediate the CSR–safety behavior link.*

### 2.6. Moderation of Moral Identity in the Corporate Social Responsibility–Job Stress Link

As previously stated, the claim that activities rooted in CSR could diminish job stress experienced by employees seems plausible. However, it could be overly simplistic to treat this relationship as a universal given [6,8]. The present inquiry proposes that the correlation between CSR and job stress could vary, contingent on the attributes of employees and the contexts within the organization. Utilizing value congruence theory, which asserts that an individual’s values should correspond with their organization’s values [65,66], this study proposes that a member’s moral identity could serve as a positive moderator in the corporate social responsibility–job stress connection.

Value congruence theory contends that the alignment between an individual member’s values and their organization’s values significantly influences their perceptions and attitudes in the workplace [65,66]. According to Hoffman and his colleagues [67], the extent of value congruence between a member and his or her company plays a mediating role in the link between transformational leadership and team effectiveness. As employees tend to proactively find meaning and understanding in their experiences at work, the value alignment between them and their organizations is vital for shaping their perceptions, attitudes, and behaviors [65,66]. Consequently, it is essential to consider value congruence when exploring the relationship between CSR activities and job stress. Thus, employees may interpret and assign meaning to their organization’s corporate social responsibility, relying on their values. This interpretative process can lead to diverse employee reactions to the social responsibilities undertaken by companies, depending on the extent of value congruence between a member and his or her firm [65,66,68].

More specifically, the influence of CSR initiatives on employee job stress may be affected by their moral identity level. Moral identity pertains to an individual’s self-concept as a moral being, significantly impacting their value system and self-identity [69,70]. Given the inherent moral aspects of CSR practices, employees’ moral identity might play a pivotal role in determining their responsiveness to CSR activities. Those with a high moral identity level may exhibit increased interest in their company’s socially responsible endeavors and be more sensitive to the effects of such activities [68,70]. When a company excels in fulfilling its social responsibilities, these employees are more likely to perceive an alignment between their own values and those they perceive as the values of the organization. In this scenario, individuals with a high moral identity are more likely to appreciate CSR activities and be more responsive, resulting in a sense of pride and positive social self-perception when their company successfully fulfills its perceived social responsibilities. This sense of pride and positive self-image can lead to reduced job stress levels [68,70]. However, individuals with a high moral identity may also experience greater disappointment than those with a low moral identity if their organization fails to execute its social responsibilities effectively. This disappointment can increase job stress levels, as these employees may feel that their values are not in harmony with those of their organization. Therefore, the relationship between CSR activities and job stress will likely intensify among those with a high moral identity [68,69,70].

Conversely, individuals with low moral identities may not exhibit a strong interest in their company’s social responsibilities. Their level of morality does not significantly impact their identity formation process. They generally do not regard moral values and ethics as crucial factors and seldom integrate them into their decision-making. Consequently, they are less sensitive to their organization’s moral conduct, and the types or extent of corporate social responsibility may not significantly influence their job stress levels. In situations where a member’s moral identity is low, the stress-reducing effect of corporate social responsibility on job stress may diminish.

**Figure 1:**
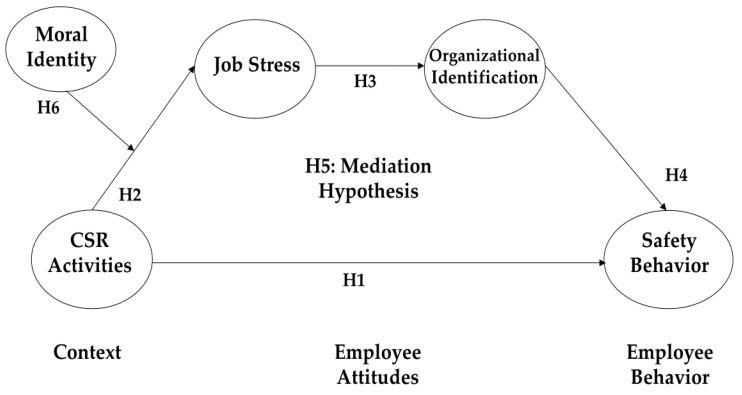
Theoretical model.

Based on the arguments presented, it can be inferred that the influence of CSR on job stress may depend on an individual employee’s moral identity level. Moral identity plays a vital role in positively moderating the corporate social responsibility–job stress link (see Figure 1).

**Hypothesis 6:**  *A member’s moral identity may positively moderate the corporate social responsibility–job stress link.*

## 3. Method

### 3.1. Participants and Procedures

To empirically explore the theoretical construct, survey responses were procured from South Korean employees over the age of 20, utilizing an online survey mechanism. The investigation collaborated with a South Korean research establishment, noted for its comprehensive aggregation of Korean panel members. In a bid to attenuate any bias within the sample, the research organization adopted a strategy of random participant selection.

The decision to focus on South Korean workers in this study is based on several important considerations. South Korea’s rapid economic growth, coupled with its unique cultural values and work environment, makes it an interesting and relevant setting for examining the relationship between CSR and safety behavior [71,72]. South Korea’s work culture is characterized by long working hours, hierarchical relationships, and a strong emphasis on group harmony [71]. These factors may influence how employees perceive and respond to their organization’s CSR initiatives, particularly those related to workplace safety. Furthermore, there is a growing need for more studies on CSR and employee behavior in non-Western contexts to enhance the generalizability of findings in this field [73]. By focusing on South Korean workers, this study contributes to the understanding of CSR and safety behavior in a cultural context that has been relatively understudied, thus expanding the scope of research in this area.

To curtail the potential impact of single-source bias, the data collection process was distributed across three distinct intervals, each separated by four to five weeks. The use of an online survey system facilitated the consistent tracking of respondents and ensured continuity of participant engagement through the first, second, and third data acquisition stages. There was an interval of six to seven weeks separating the primary and secondary surveys, while a span of seven to eight weeks was observed between the secondary and final data acquisition phases.

The research organization employed stratified random sampling as a method for participant recruitment, aiming to reduce the likelihood of sampling bias. This approach entails the selection of a random sample from each stratum, and through stratified random sampling, the probability of biases arising from varied employee characteristics (e.g., gender, age, position, educational background, and industry classification) influencing the outcomes was attenuated. The research organization monitored the respondents via multiple online mechanisms, ensuring the continuity of participants across all three data collection time points.

Experts from the research organization approached potential survey participants, inviting voluntary participation in the study, guaranteeing that responses would be treated with confidentiality and exclusively used for research-related goals. The organization also ascertained that informed consent and ethical compliance were secured from all individuals who agreed to participate and contribute their responses. Participants received a small monetary incentive from the research organization.

In the first round, 451 employees completed the survey; this figure was reduced to 313 employees in the second round, and it further dwindled to 237 employees in the third round. After discounting for incomplete or missing data, responses from 236 individuals who fully participated in all three survey periods (response rate: 52.33%) were deemed suitable for analysis. The sample size was derived from guidelines stipulated by prior research.

To determine the appropriate sample size for our study, we conducted an a priori power analysis using G*Power 3.1.9.7 [74]. We specified a desired power level of 0.80, which is the conventionally accepted level in social science research [75]. The power level of 0.80 indicates an 80% probability of detecting a true effect and a 20% probability of committing a Type II error (i.e., failing to reject a false null hypothesis). Although researchers can set higher power levels, such as 0.90 or 0.95, these levels require larger sample sizes and may not always be feasible due to resource constraints [74]. In addition to the power level, we set the significance level (α) at 0.05, which is the most commonly used level in social science research [75]. The significance level represents the probability of committing a Type I error (i.e., rejecting a true null hypothesis). By setting α at 0.05, we aimed to balance the risks of Type I and Type II errors. Based on these parameters, and assuming a medium effect size (f^2^ = 0.15) for our structural equation model, the power analysis indicated that a minimum sample size of 204 would be required to achieve a power of 0.80. Our final sample size of 236 exceeded this minimum requirement, ensuring that our study had sufficient power to detect the hypothesized relationships.

The sample size for this study was determined based on the guidelines and recommendations from previous research in the field. According to Kline [76], a sample size of at least 200 is considered adequate for structural equation modeling (SEM) analyses, as it provides sufficient statistical power to detect medium-sized effects. Furthermore, Boomsma [77] suggests that a minimum sample size of 200 is needed to obtain reliable estimates in SEM.

In line with these recommendations, several studies examining the relationship between CSR and employee outcomes have utilized sample sizes similar to or smaller than the one employed in the current study. For example, Rupp et al. [70] investigated the impact of employees’ perceptions of CSR on organizational citizenship behavior and employee engagement using a sample of 245 employees. Similarly, De Roeck et al. [78] examined the relationship between CSR perceptions and employee outcomes, such as organizational identification and job satisfaction, with a sample of 181 employees. Moreover, a review of the CSR literature by Aguinis and Glavas [1] found that the median sample size for employee-level CSR studies was 221, further supporting the adequacy of the sample size used in the present study. Given these guidelines and the sample sizes employed in comparable studies, we believe that our sample size of 236 employees is sufficient to detect meaningful relationships between the variables of interest and to ensure the reliability of our SEM analyses.

This study employed a quantitative research methodology, using a three-wave time-lagged survey design to collect data from South Korean employees. The time-lagged design was chosen to minimize common method variance and to establish temporal precedence among the variables [79]. Data were collected at three time points, each separated by a 4–5-week interval, to reduce the potential influence of common method bias and to allow for the examination of causal relationships [80,81].

Moreover, applying a 3-wave time-lagged research design provides several advantages over cross-sectional designs, particularly in addressing potential issues associated with the latter approach. One such justification for employing a three-wave time-lagged design lies in its ability to establish temporal precedence, which is crucial in determining the causal relationships among variables. Cross-sectional designs, which collect data at a single point in time, often fail to accurately establish causality due to the inability to discern the temporal ordering of the variables. In contrast, the three-wave time-lagged design allows researchers to examine the temporal sequence of events by collecting data at multiple time points, thereby enhancing the potential to infer causal relationships among the variables under study.

Another significant advantage of the three-wave time-lagged approach is its capacity to address the issue of common method variance (CMV) that often plagues cross-sectional designs. CMV arises when the measurement of predictor and criterion variables occurs simultaneously, potentially leading to inflated relationships between the variables due to shared method variance. By collecting data at three distinct time points, the three-wave time-lagged design minimizes the risk of CMV, as the predictor and criterion variables are measured at different intervals. This temporal separation mitigates the potential influence of method variance on the observed relationships, thereby providing a more accurate representation of the true associations among the variables. Consequently, the three-wave time-lagged data collection methodology offers a robust and reliable alternative to cross-sectional designs, addressing several inherent limitations and enhancing the validity of research findings.

### 3.2. Measures

Participants were requested to assess their organization’s CSR level and the level of their moral identity and provide their demographic characteristics at Time Point 1. Data on employees’ job stress and organizational identification were collected at Time 2, and safety behaviors were surveyed at Time 3. All study variables were evaluated using a multi-item 5-point Likert scale (ranging from 1 = strongly disagree to 5 = strongly agree). The internal consistency of the variables was measured using Cronbach’s α values.

#### 3.2.1. CSR (Time Point 1, Gathered from Employees)

We assessed employees’ perceptions of their organization’s CSR activities using a 12-item scale developed by Turker [82]. This scale was chosen because it encompasses multiple stakeholder dimensions of CSR, including employees, customers, the environment, and society, which aligns with the multifaceted nature of CSR [1]. The scale has demonstrated good psychometric properties in previous studies (e.g., Farooq et al. [83]; Kim and Choi [68]; Kim and Kim [27]) and has been widely used in CSR research, allowing for comparability with other studies. Sample items include “My organization supports non-governmental organizations working in problematic areas” and “My organization contributes to campaigns and projects that promote the well-being of society.” Responses were measured on a 5-point Likert scale (1 = strongly disagree, 5 = strongly agree). The scale’s reliability in our sample was α = 0.92.

#### 3.2.2. Moral Identity (Time Point 1, Gathered from Employees)

To measure employees’ moral identity, we used a 5-item scale adapted from Aquino and Reed [69]. This scale has been widely used and validated in numerous studies examining the role of moral identity in various organizational contexts (e.g., Rupp et al. [70]; Shao et al. [84]; Skarlicki et al. [85]). The scale assesses the extent to which moral traits (e.g., being caring, compassionate, fair, friendly, generous, helpful, hardworking, honest, and kind) are central to an individual’s self-concept. Participants were asked to visualize a person with these characteristics and then indicate their agreement with statements such as “It would make me feel good to be a person who has these characteristics” and “Having these characteristics is an important part of who I am.” The scale has demonstrated high reliability and validity in previous research [86,87], and its use has been supported by a comprehensive review of the moral identity literature [84]. In the current study, Cronbach’s alpha for the moral identity scale was 0.738, indicating good internal consistency.

#### 3.2.3. Job Stress (Time Point 2, Gathered from Employees)

We assessed employees’ job stress using a 6-item scale adapted from previous works [88,89]. This scale has been widely used in organizational research and has demonstrated good psychometric properties (e.g., Jamal and Baba [90]; Tucker, Sinclair, and Thomas [91]). The scale items reflect common sources of job stress, such as workload, time pressure, and role ambiguity, which are theoretically linked to employee well-being and performance [92]. Sample items include “At work, I felt stressed during the last 30 days” and “At work, I felt anxious during the last 30 days”. The scale’s reliability in our sample was α = 0.895.

#### 3.2.4. Organizational Identification (Time Point 2, Gathered from Employees)

We measured employees’ organizational identification using a 6-item scale developed by Mael and Ashforth [54]. This scale is grounded in social identity theory and has been extensively used in organizational research (e.g., Riketta [55]; van Dick et al. [93]). The scale assesses the extent to which employees define themselves in terms of their membership in the organization and has demonstrated good construct validity [54]. Sample items include “When someone criticizes my organization, it feels like a personal insult” and “My organization’s successes are my successes.” Responses were measured on a 5-point Likert scale (1 = strongly disagree, 5 = strongly agree). The scale’s reliability in our sample was α = 0.853.

#### 3.2.5. Safety Behavior (Time Point 3, Gathered from Employees)

We assessed employees’ safety behavior using a 6-item scale developed by Neal and Griffin [94]. This scale measures two dimensions of safety behavior: safety compliance (adhering to safety rules and procedures) and safety participation (voluntarily participating in safety activities). The scale has been widely used in safety research and has demonstrated good psychometric properties (e.g., Christian et al. [29]; Inness et al. [95]). The scale items reflect key aspects of safety behavior that are theoretically linked to workplace safety outcomes [96]. Sample items include “I use the correct safety procedures for carrying out my job” (safety compliance) and “I voluntarily carry out tasks or activities that help to improve workplace safety” (safety participation). Responses were measured on a 5-point Likert scale (1 = strongly disagree, 5 = strongly agree). The scale’s reliability in our sample was α = 0.917.

#### 3.2.6. Control Variables

To minimize estimation bias and enhance the validation of these findings, we controlled for the potential influence of gender, education, position, and employment tenure on safety behavior [94]. These variables were assessed at Time Point 1.

### 3.3. Analytical Strategy

In order to evaluate the validity of our investigative model, we employed SPSS 26.0 and Amos 26.0 software (IBM Corp., Armonk, NY, USA) to carry out a range of statistical analyses. Initially, the demographic traits of the sample group were examined utilizing frequency analysis. Subsequently, the uniqueness of the measures was validated through the use of confirmatory factor analysis. The study employed Pearson correlation analysis to probe the associations between the variables under study. Hypotheses were put to the test through the application of structural equation modeling (SEM) using Amos 26.0. We followed the advice provided by Anderson and Gerbing [97] and adopted a two-step process involving (1) measurement and (2) structural modeling. Confirmatory factor analysis was implemented to validate the measurement model. Following this, the AMOS 26.0 software was used to conduct a moderated mediation model analysis employing the maximum likelihood (ML) estimator to evaluate the structural model via SEM. SEM is a multivariate statistical technique that allows for the simultaneous examination of multiple relationships among observed and latent variables [76]. We followed Anderson and Gerbing’s [97] two-step approach, first assessing the measurement model and then examining the structural model. The measurement model was evaluated using confirmatory factor analysis (CFA) to ensure the reliability and validity of the measures. The structural model was then assessed to test the hypothesized direct, indirect, and moderated relationships among the study variables.

In order to determine the acceptability of diverse model fit indices, an array of goodness-of-fit indices, including the comparative fit index (CFI), the Tucker–Lewis index (TLI), and the root mean square error of approximation (RMSEA) were brought into use. Existing research recommends that CFI and TLI values above 0.90 and an RMSEA value below 0.06 are acceptable thresholds [98].

Finally, a bootstrapping analysis was executed to evaluate the significance of the indirect mediation impact between corporate social responsibility activities and safety behavior using a 95% bias-corrected confidence interval (CI). The results of this analysis reveal if the indirect effect holds statistical significance at a 0.05 level, providing the CI does not encompass zero (0) [99].

## 4. Results

### 4.1. Descriptive Statistics

Table 1 presents the means, standard deviations, and correlations among the study variables. Employees’ perceptions of their organization’s CSR activities were positively correlated with their safety behavior (r = 0.46, *p* < 0.01) and organizational identification (r = 0.52, *p* < 0.01), and negatively correlated with job stress (r = −0.25, *p* < 0.01). Job stress was negatively correlated with organizational identification (r = −0.18, *p* < 0.01) and safety behavior (r = −0.07, *p* < 0.05). Organizational identification was positively correlated with safety behavior (r = 0.37, *p* < 0.01).

### 4.2. Measurement Model

Confirmatory factor analyses (CFAs) were executed to evaluate the goodness-of-fit of the measurement model. The model encompassed five research constructs: corporate social responsibility (CSR), job stress, organizational identification, moral identity, and safety behavior. The five-factor structure demonstrated a good fit (χ^2^ (df = 214) = 390.912; CFI = 0.944; TLI = 0.934; RMSEA= 0.059). Following this, a sequence of χ^2^ difference tests were carried out, comparing the four-factor model with various alternative models, including four-factor (χ^2^ (df = 218) = 716.275; CFI = 0.842; TLI = 0.816; RMSEA= 0.099), three-factor (χ^2^ (df = 221) = 1130.872; CFI = 0.711; TLI = 0.669; RMSEA = 0.132), two-factor (χ^2^ (df = 223) = 1311.424; CFI = 0.654; TLI = 0.608; RMSEA = 0.144), and one-factor (χ^2^ (df = 224) = 1720.280; CFI = 0.524; TLI = 0.463; RMSEA = 0.169) models. The results of the χ^2^ difference tests suggest that the five-factor model exhibits the most optimal fit compared to the alternative models, indicating that the five constructs possess satisfactory discriminant validity.

### 4.3. Structural Model

In this investigation, a moderated mediation model was established to scrutinize the association between CSR activities and workplace safety behavior. The model consisted of: (1) a mediating structure, wherein the connections between CSR and safety behavior were sequentially mediated by employee job stress and organizational identification; and (2) a moderating structure, wherein the relationship between CSR activities and job stress was moderated by an employee’s moral identity. To assess the probability of multi-collinearity bias between CSR and moral identity, variance inflation factors (VIF) and tolerances were calculated [100]. The outcomes indicated that both corporate social responsibility and moral identity were not significantly influenced by the problem of multi-collinearity because VIF scores (CSR = 1.028, moral identity = 1.028) were below 10 and tolerance scores were above 0.2 (CSR = 0.972, moral identity = 0.972).

### 4.4. Results of Mediation Analysis

To ascertain the most fitting model, structural equation model analyses were carried out, and a χ^2^ difference test compared our hypothesized model with the alternative (i.e., a full mediation model and a partial mediation model). The full mediation model was identical to the partial mediation model, except for the direct path connecting CSR activities and safety behavior. Both models exhibited acceptable fit indices. The full mediation model (χ^2^ = 432.488 (df = 226), CFI = 0.925, TLI = 0.909, and RMSEA = 0.062) and the partial mediation model (χ^2^ = 410.500 (df = 225), CFI = 0.933, TLI = 0.917, and RMSEA = 0.059) demonstrated satisfactory fit. However, the χ^2^ difference test comparing the two models (Δχ^2^ [1] = 21.988, *p* < 0.01) indicated that the partial mediation model was superior, suggesting that CSR would affect safety behavior both ‘directly’ and ‘indirectly’ (through the sequential mediating effect of job stress and organizational identification) rather than solely exerting a direct influence on it.

**Figure 2:**
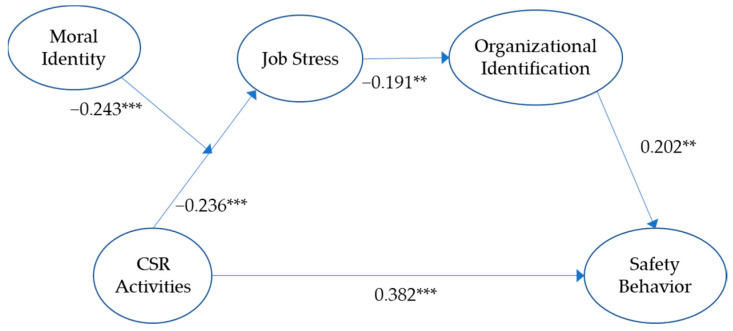
Results of the final model (** *p* < 0.01, *** *p* < 0.001. All values are standardized).

All control variables (gender, position, tenure, and education level) of this research model were not significant. All hypotheses were supported by the research model, including Hypothesis 1, that corporate social responsibility enhances safety behavior (β = 0.382, *p* < 0.001); Hypothesis 2, that CSR activities decrease job stress (β = −0.236, *p* < 0.001); Hypothesis 3, that job stress decreases organizational identification (β = −0.191, *p* < 0.01); and Hypothesis 4, that organizational identification increases safety behavior (β = 0.202, *p* < 0.01) (See Table 2 and Figure 2).

Support for Hypothesis 1 was evident from the significant path coefficient value between CSR activities and safety behavior in the partial mediation model. This finding is consistent with the improved model fit indices of the partial mediation model compared to the full mediation model. Consequently, we infer that CSR activities influence safety behavior both directly and indirectly through various mediators (e.g., job stress and organizational identification). The results of the chi-squared difference test between the full and partial mediation models, along with the significant path coefficient value, provide support for Hypothesis 1.

### 4.5. Analysis Results of Sequential Mediation

In order to validate Hypothesis 5—which postulates that job stress and organizational identification could potentially act as sequential mediators between CSR and safety behavior—the research adopted a bootstrapping approach utilizing a substantial sample of 10,000. Bootstrapping is a statistical resampling technique used extensively in testing hypotheses, especially within the framework of structural equation modeling (SEM) and other complex statistical analyses. Its necessity, particularly when testing specific hypotheses, such as the fourth hypothesis in the given context, could be attributed to several reasons related to the nature of the hypothesis itself and the statistical requirements it entails [99]. While the specific details of the fourth hypothesis are not provided, the rationale for employing bootstrapping exclusively for this hypothesis can generally be understood through the following considerations. The fourth hypothesis involves testing mediation effects, which require the estimation of indirect effects. Bootstrapping is favored in such instances because it does not impose the assumption of normality on the sampling distribution of the indirect effect, making it a robust method for assessing the significance of these effects [99]. In addition, if the data related to the fourth hypothesis exhibit non-normal distribution or if the sample size is relatively small, bootstrapping can provide more accurate confidence intervals and significance tests by relying on the empirical distribution of the sample rather than theoretical distribution assumptions [99].

This procedure aligned with the methodology delineated by Shrout and Bolger [99]. For the indirect effect emanating from job stress and organizational identification to be deemed statistically significant, the 95% bias-corrected confidence interval (CI) pertaining to the average indirect effect must eschew the inclusion of the value zero, as per the guidance of Shrout and Bolger [99].

Further, to meticulously assess the sequential mediating role of job stress and organizational identification linking CSR to safety behavior (as posed in Hypothesis 5), the investigation employed a bootstrapping technique with the aforementioned sample size. The statistical significance of this indirect mediation effect is affirmed when the 95% bias-corrected CI pertaining to this average effect precludes the value zero. The findings from this analysis yielded a CI that distinctly did not encompass zero (95% CI = [0.001, 0.025]), conclusively signifying that the sequential mediating role of job stress and organizational identification holds statistical weight. This not only solidifies the claims made in Hypothesis 5 but also further accentuates the intricate connections. The comprehensive delineation of the direct, indirect, and total effects of the trajectories originating from CSR to safety behavior is meticulously presented in Table 3.

### 4.6. Results of Moderation Analysis

**Figure 3:**
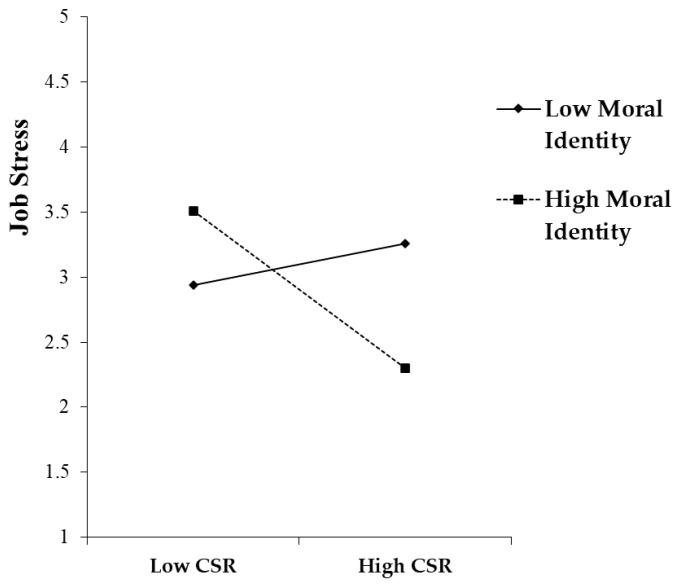
The moderating effect of moral identity in the CSR–job stress link.

In order to examine the moderating influence of moral identity on the relationship between CSR and job stress, this investigation formulated a moderated mediation model by creating an interaction term with a mean-centering method. This facilitated the efficient estimation of interaction terms while mitigating multicollinearity among the variables. The coefficient of the interaction term was found to be statistically significant (β = −0.243, *p* < 0.001), implying that moral identity moderates the CSR–job stress connection by amplifying the decreasing effect of CSR on job stress. This outcome provided support for Hypothesis 6, as an elevated level of moral identity intensifies the decreasing effect of CSR on employee job stress. A graphical depiction of these outcomes can be found in Figure 3.

## 5. Discussion

The present study contributes to the literature on corporate social responsibility (CSR) and employee outcomes by examining the relationship between CSR and safety behavior, as well as the underlying mechanisms and boundary conditions of this relationship. Our findings provide support for the hypothesized relationships and offer insights into how CSR can influence employee safety behavior through the sequential mediation of job stress and organizational identification, as well as how moral identity moderates the relationship between CSR and job stress [1,8,101].

Specifically, our finding that CSR directly enhances employee safety behavior aligns with research that has linked CSR to improved safety outcomes. For example, in a study of the oil and gas industry, Agan et al. [102] found that employees’ perceptions of their organization’s CSR were positively related to their safety participation and compliance. Similarly, Hofmann et al. [38] demonstrated that employees’ perceptions of their organization’s commitment to safety, a form of CSR, were positively associated with safety citizenship behavior. Our study extends these findings by examining the impact of CSR on safety behavior in the South Korean context, thus contributing to the generalizability of this relationship across different cultural settings.

Moreover, our study sheds light on the mechanisms underlying the CSR–safety behavior relationship by identifying job stress and organizational identification as sequential mediators. These findings are consistent with research that has linked CSR to reduced job stress [103,104] and increased organizational identification [21,105]. However, our study goes beyond these works by demonstrating the sequential nature of these mediating effects, providing a more nuanced understanding of how CSR ultimately influences employee safety behavior. This sequential mediation model aligns with the findings of Wang et al. [106], who found that CSR influenced employee engagement through the sequential mediation of organizational identification and perceived organizational support. In addition, our study extends these findings by examining the relationship between CSR and organizational identification in the context of safety behavior, suggesting that CSR can foster employees’ sense of belonging and alignment with their organization, which in turn promotes safety compliance and participation. Also, our study contributes to this body of research by examining the mediating role of job stress in the relationship between CSR and safety behavior, suggesting that CSR can reduce job stress, which in turn promotes safety compliance and participation.

The moderating role of moral identity in the relationship between CSR and job stress identified in our study is also consistent with previous research. Our findings align with the work of Rupp et al. [70], who found that employees’ moral identity moderated the relationship between their perceptions of CSR and various work outcomes, such that the positive effects of CSR were stronger for employees with a high moral identity. Similarly, Gao and He [107] found that moral identity moderated the relationship between CSR and employee well-being, with the positive effects of CSR being more pronounced for employees with a high moral identity. Our study extends this line of research by demonstrating that moral identity can also influence the relationship between CSR and job stress, with the negative effect of CSR on job stress being stronger for employees with higher moral identity. This finding highlights the importance of considering individual differences in moral identity when examining the impact of CSR on employee outcomes.

While our findings are largely consistent with previous research, it is important to acknowledge the limitations of our study and consider how our results may be bounded by the specific cultural context in which the research was conducted. As noted earlier, the South Korean context, characterized by high collectivism and power distance [108], may have influenced the relationships observed in our study. Future research could explore these relationships in other cultural settings to assess the generalizability of our findings and investigate potential cultural differences in the mechanisms linking CSR to employee outcomes [21,109].

Furthermore, while our study focused on safety behavior as an outcome of CSR, it is important to consider how CSR may influence other types of employee behavior and well-being. For example, Glavas and Piderit [103] found that employees’ perceptions of CSR were positively related to their creative involvement, while Meynhardt et al. [110] demonstrated that CSR could enhance employees’ sense of meaning and purpose at work. Future research could explore a broader range of employee outcomes and investigate the potential mediating and moderating factors that shape these relationships.

In conclusion, our study contributes to the literature by providing a more comprehensive understanding of how CSR influences employee safety behavior, identifying key mediating and moderating factors that shape this relationship. While our findings are largely consistent with previous research, we extend this literature by demonstrating the sequential nature of the mediating effects and the specific moderating role of moral identity in the South Korean context. Future research could build upon these findings by exploring these relationships in other cultural contexts and examining a broader range of employee outcomes.

### 5.1. Theoretical Implications

The present study offers a noteworthy addition to the existing body of CSR literature from a theoretical perspective. First, it delves into the impact of corporate social responsibility on employees’ behavior. While previous research has predominantly focused on the influence of CSR on members’ attitudes and perceptions, such as organizational identification, job satisfaction, organizational trust, and perceived organizational support, insufficient attention has been given to its effects on behavior [6,8,9]. Although members’ attitudes and perceptions are crucial outcomes for organizations, they ultimately manifest as behaviors. Members’ behaviors are likely to exert a more significant impact on macro-level performances, such as financial performance, than do employees’ attitudes and perceptions [6,8,9,27]. Thus, this study’s emphasis on examining the impact of corporate social responsibility on safety behavior by employees is anticipated to contribute to both the corporate social responsibility and safety behavior literature.

Second, in the context of the COVID-19 pandemic, the importance of employees’ safety behavior has been underscored as a vital precursor to consumer and company safety during major health-related crises [30]. Despite its relevance, there has been limited research on safety behavior. Consequently, this study is valuable for both scholars and practitioners seeking a comprehensive understanding of the impact of CSR on safety behavior [27,30].

The third aim of this research was to uncover the mediating processes in the corporate social responsibility–safety behavior link by identifying mediating and moderating variables. It is posited that CSR practices may alleviate employee job stress, subsequently bolstering their organizational identification and ultimately resulting in enhanced safety behavior. Thus, fulfilling CSR obligations may significantly contribute to organizational outcomes by positively affecting employees’ job stress and identification levels. This paper’s thorough elucidation of the mechanism through which CSR activities improve organizational outcomes represents a valuable contribution to the expansion and deepening of the CSR literature.

The last objective of this study was to demonstrate the critical moderating role of individual members’ moral identity in the connection between CSR activities and job stress. The extent to which an organization satisfies its responsibility for society by mitigating employee job stress may not be consistent among all employees but is contingent upon each employee’s moral identity [55,56]. For example, an employee who does not perceive their company’s ethical conduct (i.e., CSR initiatives) as significantly impacting their job stress formation may not experience a considerable reduction in stress levels, regardless of the firm’s CSR activities effectiveness [51,52,54]. In contrast, an individual whose moral identity is paramount to his or her self-concept may react with escalating job stress levels if their organization neglects CSR. We believe that the effort to explore the mediating variables and contingent factors in the CSR–safety behavior link constitutes a valuable contribution to the existing literature.

### 5.2. Practical Implications

The current research offers practical applications for organizations. First, in line with the prior literature, upper management must understand that CSR initiatives are not merely passive measures to satisfy social responsibilities that are perceived as costs [13,14]. Addressing social obligations can alleviate employee job stress, thereby fostering increased employee loyalty toward the organization. The heightened organizational identification enhances decision-making quality and promotes positive behaviors among members, including safety behavior. The execution of CSR activities can substantially improve employee safety behavior by reducing job stress levels and strengthening organizational identification. Given the significance of safety behavior in the workplace, embracing CSR represents a valuable investment to augment various firm outcomes. This assertion is supported by earlier research that demonstrated how CSR improves organizational results by elevating members’ perceptions and attitudes within an organization.

Second, this research provides guidance to top management teams and managers seeking to evaluate the influence of CSR on organization members. Specifically, to ascertain the efficacy of CSR initiatives, managers must examine members’ job stress and organizational identification levels. The findings of the sequential mediation analysis in this study suggest that job stress and organizational identification levels successfully mediate the favorable effect of CSR on safety behavior. Consequently, to gauge the relative effectiveness of CSR activities, managers must review and oversee job stress and organizational identification levels among employees. Employee job stress and organizational identification are vital criteria for determining whether the positive effects of corporate social responsibility are materializing in the workplace.

Third, the study highlights the necessity of addressing job stress among employees, as it mediates CSR and employee safety behavior. Consequently, those in leadership positions must proactively identify workplace stressors and implement suitable interventions to alleviate their impact. Such measures may encompass equipping employees with essential resources to manage work-related stress, nurturing a supportive work environment, and encouraging work–life balance.

Lastly, corporate managers must recognize that employees’ responses to CSR activities vary, and individual differences should be considered. In particular, this study highlights the importance of individual members’ degree of moral identity in determining the impact of CSR activities undertaken by firms. Even when a company meets its ethical obligations, members with a low moral identity would not be significantly influenced by the organization’s ethical endeavors. On the contrary, members with a high moral identity could be highly receptive to the company’s CSR efforts. The study highlights the role of employees’ moral identity as a positive moderator in the relationship between CSR and job stress. This suggests that organizations should invest in fostering a solid moral identity among employees, as it can amplify the positive effects of CSR initiatives on job stress reduction. Strategies for promoting moral identity might include incorporating ethical training programs, establishing clear ethical guidelines, and encouraging ethical behavior within the workplace.

### 5.3. Limitations and Suggestions for Future Research

This manuscript acknowledges several limitations that warrant further investigation. Firstly, the research inadequately considers the cultural disparities between Eastern and Western societies related to their perception of an organization’s CSR endeavors. Prior research indicates that Western cultures prioritize socially enforced responsibilities, encompassing CSR initiatives, and their members are more inclined to acknowledge social commitments [111,112]. Since this research gathered data solely from South Korean employees, prudence is essential when extrapolating the findings to other cultural settings [111,112]. While CSR’s essence remains universal, South Korean employees may exhibit distinctive reactions to CSR compared to their Western counterparts. Subsequent studies should thoroughly investigate this factor.

Second, the present research could not employ an objective metric for assessing the scope of CSR practices; instead, it relied exclusively on employee subjective evaluations. Although prior studies on corporate social responsibility have posited that subjective indicators, such as a member’s perception of corporate social responsibility, can effectively represent the actual occurrences of corporate social responsibility [6,8], future research should incorporate both objective and subjective measures to analyze the contrasting impacts of each.

Third, this research insufficiently examined the potential discriminatory influence of diverse aspects of CSR practices [21,27]. As previously highlighted, the goals of CSR can range widely, encompassing CSR efforts directed towards members, customers, communities, government, and the environment. Nonetheless, the current paper only assessed four sub-components of corporate social responsibility (namely, corporate social responsibility for the environment, community, employees, and customers). Consequently, participants may exhibit varying responses depending on the intended beneficiary of the CSR activities. For instance, Farooq and his colleagues [21] discovered that CSR practices could elicit divergent perceptions among employees, depending on whether the initiatives were classified as internal or external CSR activities. This matter should be more comprehensively explored in subsequent investigations.

Fourth, while our time-lagged design helps to establish temporal precedence among the variables, it does not allow for strong causal inferences. Future research could employ experimental or quasi-experimental designs to provide more robust evidence of causality in the relationships between CSR, job stress, organizational identification, moral identity, and safety behavior.

Fifth, although employing a three-wave, time-lagged data collection approach mitigates some limitations endemic to cross-sectional designs, unobserved confounding factors may still influence the relationships between CSR, job stress, employee organizational identification, moral identity, and safety behavior. To fortify the robustness of the findings, future investigations could adopt advanced statistical techniques, such as structural equation modeling or propensity score matching, which would account for potential confounding variables.

Sixth, the current study scrutinizes a specific set of mediators (job stress and employee organizational identification) and a single moderator (moral identity) in the relationship between CSR and safety behavior. While this offers valuable insights into the complex interplay of variables, additional mediators and moderators might further clarify the dynamics at play. Subsequent research should consider exploring other potential mediators, such as employees’ perceptions of organizational support or trust in management, and additional moderators, including organizational culture or leadership style, to achieve a more comprehensive understanding of the factors influencing the CSR–safety behavior nexus.

Seventh, it is important to acknowledge the potential limitations of this study arising from the cultural specificity of our sample. South Korean society is characterized by cultural values such as collectivism and a high power distance [108,113]. These values may influence employees’ perceptions of CSR and their subsequent attitudes and behaviors. For example, in a collectivistic culture like South Korea, employees may be more likely to prioritize group interests over individual needs, which could affect their engagement with CSR initiatives [71]. Additionally, the high power distance in South Korean culture may lead employees to feel less empowered to speak up about safety concerns or participate in safety-related CSR activities [72]. As a result, the findings of this study may not be directly generalizable to other cultural contexts, particularly those with different cultural values. Future research should examine the relationship between CSR and safety behavior in diverse cultural settings to develop a more comprehensive understanding of these relationships.

Eighth, the limitations associated with the cultural specificity of our sample highlight the importance of cross-cultural research in the CSR and safety behavior domain. Future studies should investigate the moderating role of cultural values on the relationship between CSR and employee outcomes, including safety behavior [73,114]. By examining how cultural values such as collectivism, power distance, and uncertainty avoidance influence employees’ perceptions and responses to CSR initiatives, researchers can develop a more nuanced understanding of the boundary conditions for the effectiveness of CSR in promoting safety behavior. Moreover, future research should explore the relationship between CSR and safety behavior in various cultural contexts, including both Western and non-Western settings. This will help to establish the generalizability of findings and identify potential cultural differences in the mechanisms linking CSR to employee outcomes. By conducting research in diverse cultural contexts, scholars can contribute to the development of a more comprehensive and culturally sensitive framework for understanding the impact of CSR on workplace safety.

Ninth, our study relies on self-reported data from a single source, which may raise concerns about common method variance (CMV). Although we employed a three-wave time-lagged design to mitigate the potential impact of CMV, future research could strengthen the findings by collecting data from multiple sources, such as supervisors or coworkers, and by using objective measures of safety behavior (e.g., safety records, accident reports).

Finally, our study relied on a convenience sample of South Korean employees, which may not be representative of the broader population. Future research could use probability sampling techniques to obtain more representative samples and enhance the external validity of the findings.

## 6. Conclusions

The present study contributes to the growing body of research on corporate social responsibility (CSR) and employee outcomes by examining the relationship between CSR and safety behavior, as well as the underlying mechanisms and boundary conditions that shape this relationship. Our findings demonstrate that CSR has a direct positive impact on employee safety behavior and that this relationship is sequentially mediated by job stress and organizational identification. Furthermore, we found that employees’ moral identity moderates the relationship between CSR and job stress, such that the negative impact of CSR on job stress is stronger for employees with a high moral identity.

These findings have important implications for both theory and practice. From a theoretical perspective, our study extends previous research on CSR and employee outcomes by focusing on safety behavior as a critical outcome variable. While prior studies have examined the impact of CSR on various employee attitudes and behaviors [1,8], our study is among the first to investigate the relationship between CSR and safety behavior, thus contributing to a more comprehensive understanding of the employee-related outcomes of CSR.

Moreover, by identifying job stress and organizational identification as sequential mediators in the relationship between CSR and safety behavior, our study sheds light on the psychological mechanisms through which CSR influences employee outcomes. These findings are consistent with the conservation of resources theory [44] and the social identity theory [57], which suggest that CSR can serve as a valuable resource for employees, reducing their job stress and enhancing their identification with the organization, ultimately leading to improved safety behavior.

Our study also highlights the important role of individual differences in shaping employees’ responses to CSR. By demonstrating that moral identity moderates the relationship between CSR and job stress, our findings suggest that the impact of CSR on employee outcomes may vary depending on employees’ personal values and beliefs. This is consistent with the person–organization fit theory [115], which posits that the alignment between individual and organizational values can influence employee attitudes and behaviors.

From a practical standpoint, our findings underscore the importance of CSR as a strategic tool for promoting employee well-being and safety. Organizations should prioritize CSR initiatives not only for their positive impact on society and the environment but also for their potential to enhance employee outcomes. By investing in CSR, organizations can create a supportive work environment that reduces employee stress, fosters organizational identification, and encourages safe behavior.

Furthermore, our study suggests that organizations should consider employees’ individual differences when implementing CSR initiatives. Managers should be aware that employees with a high moral identity may be more responsive to CSR and may experience greater reductions in job stress as a result of CSR initiatives. Organizations may benefit from fostering a strong moral identity among their employees through ethics training, value-based leadership, and a culture that emphasizes ethical behavior.

In conclusion, our study provides new insights into the relationship between CSR and employee safety behavior, highlighting the mediating roles of job stress and organizational identification and the moderating role of moral identity. These findings contribute to the growing literature on CSR and employee outcomes and have important implications for organizations seeking to promote employee well-being and safety through CSR initiatives. Future research should build upon these findings by examining additional mediators and moderators, exploring the impact of CSR on other types of employee behavior, and investigating the boundary conditions of these relationships across different cultural and organizational contexts.

## Figures and Tables

**Table 1:** Correlation among research variables.

	Mean	S.D.	1	2	3	4	5	6	7	8
1. Gender	1.49	0.50	-							
2. Education	2.75	0.79	−0.19 **	-						
3. Tenure	7.83	7.68	−0.31 **	−0.07	-					
4. Position	2.97	1.62	−0.46 **	0.22 **	0.27 **	-				
5. CSR	3.15	0.71	−0.21 **	0.05	0.19 **	0.14 *	-			
6. MI	3.74	0.63	0.04	0.14 *	−0.05	0.09	0.17 *	-		
7. JS	2.90	0.78	0.07	−0.01	0.01	−0.02	−0.25 **	−0.13 *	-	
8. OI	3.41	0.73	−0.22 **	−0.02	0.17 *	0.20 **	0.52 **	0.29 **	−0.18 **	-
9. SB	3.68	0.66	−0.19 **	0.03	0.14 *	0.10	0.46 **	0.26 **	−0.07	0.37 **

Notes: * *p* < 0.05. ** *p* < 0.01. S.D. means standard deviation. CSR indicates corporate social responsibility; MI indicates moral identity; JS indicates job stress; OI means organizational identification; and SB means safety behavior.

**Table 2:** Results of the structural model.

Hypothesis	Path	Unstandardized Estimate	S.E.	Standardized Estimate	Supported
1	CSR → Safety Behavior	0.305	0.060	0.382 ***	Yes
2	CSR → Job Stress	−0.223	0.066	−0.236 ***	Yes
3	Job Stress → Organizational Identification	−0.155	0.057	−0.191 **	Yes
4	Organizational Identification → Safety Behavior	0.210	0.069	0.202 **	Yes
6	CSR × Moral Identity	−0.382	0.102	−0.243 ***	Yes

Notes: ** *p* < 0.01, *** *p* < 0.001. Estimate indicates standardized coefficients; S.E. means standard error; CSR means corporate social responsibility. The path from CSR to safety behavior was in the partial mediation model.

**Table 3:** Direct, indirect, and total effects of the final research model.

Model (Hypothesis 5)	Direct Effect	Indirect Effect	Total Effect
CSR → Job Stress → Organizational Identification → Safety Behavior	0.305	0.007	0.312

## Data Availability

Data are contained within the article.
